# Reporting Matters: Severe Adverse Events in Soft Tissue Sarcoma Therapy—A 30-Year Systematic Review of Placebo- and Non-Systemic-Controlled Randomized Trials

**DOI:** 10.3390/cancers17193118

**Published:** 2025-09-25

**Authors:** Rahel Aeschbacher, Bruno Fuchs, Gabriela Studer, Philip Heesen

**Affiliations:** 1 Faculty of Health Sciences and Medicine, University of Lucerne, 6002 Lucerne, Switzerland; 2 Swiss Sarcoma Network SSN, Geschäftsstelle, LUKS University Hospital, 6000 Lucerne, Switzerland; 3 LUKS Sarcoma-IPU, Department of Orthopaedics and Trauma, LUKS University Hospital, 6000 Lucerne, Switzerland; 4 Department of Radiation Oncology, LUKS University Hospital, 6000 Lucerne, Switzerland; 5 Medical Faculty, University of Zurich, 8032 Zurich, Switzerland

**Keywords:** soft-tissue sarcoma, systemic therapy, chemotherapy, adverse events, toxicity, CTCAE, randomized controlled trials, meta-analysis, reporting standards

## Abstract

**Background:** Systemic therapy for soft tissue sarcoma (STS) provides modest survival benefit but carries clinically relevant toxicity. Published trials report adverse events (AEs) of varying quality and extension. Poor toxicity reporting hampers balanced risk–benefit appraisal. **Methods:** A PRISMA-2020 systematic review was registered in PROSPERO CRD420251087366. PubMed, CENTRAL, and Google Scholar were searched from 16 December 2024 to 16 April 2025 for randomized controlled trials (RCTs) evaluating chemotherapy, kinase inhibitors, or immune checkpoint inhibitors in STS. AE terms were harmonized to CTCAE v5.0; event rates were normalized to patients evaluable for safety. Pooled proportions used DerSimonian–Laird random-effects models; between-group comparisons employed unpaired *t*-tests. Risk of bias (RoB 2) was assessed with the Cochrane RoB 2 tool. **Results:** Ten RCTs (1079 treated, 979 control patients; 1994–2024) met the inclusion criteria, although two lacked sufficient presentation of toxicity data and seven failed to report parallel control-arm AEs. Pooled normalized incidences for treated patients were as follows: grade ≥ 3 hematological AEs, 17% (95% CI 14–20); severe gastrointestinal AEs, 9% (8–11); and grade 4 AEs, ≤6%. Anthracycline-based and kinase-inhibitor regimens displayed comparable composite grade ≥ 3 burdens (58% vs. 84%, *p* = 0.64). Between-study heterogeneity was considerable for gastrointestinal and hematological events (*I*^2^ > 60%), driven by differing AE scales and denominators. Late-effect toxicities (cardiac, hepatic, neurological, and nephrological) were rarely reported, occurring in <1% of the patients. Across the three RCTs with control-arm data, experimental therapy increased common grade 3 AEs by 4–12 percentage points (*p* = 0.001). RoB 2 flagged serious concerns in 4/10 trials. **Conclusions:** Severe AEs in STS systemic therapy are moderately frequent; while the toxicity spectrum differs across drug classes (e.g., hematological for anthracyclines vs. neuropathic or fatigue-related for agents such as eribulin), the aggregate burden of severe AEs has not been lower for newer agents. Confidence in these estimates is limited by incomplete and non-standardized AE reporting. Future sarcoma trials must adopt CTCAE v5.0, specify explicit safety denominators, and publish full AE matrices to enable high-certainty risk–benefit assessment.

## Simple Summary

Chemotherapy and newer targeted drugs are often used to treat soft tissue sarcoma, a rare malignant tumour of mesenchymal origin. We looked at every randomized clinical trial of systemic therapy for soft tissue sarcoma published in the past 30 years and re-analyzed the safety information. After standardizing the data, we found that about 1 in 6 patients developed severe drops in white-blood-cell counts and 1 in 10 suffered severe stomach- or bowel-related problems. Life-threatening side effects occurred in fewer than 1 in 20 patients. Newer drugs were no safer than traditional chemotherapy. However, only 50% of the selected studies reported side effects extensively and 3/8 used different grading systems, so today’s risk estimates are still uncertain. Our findings show that serious but manageable side effects are common, and they highlight the urgent need for future sarcoma trials to report safety data consistently and transparently. Clearer reporting will help patients make better-informed treatment choices and allow doctors to prepare more effective supportive care.

## 1. Introduction

Soft tissue sarcomas (STS) encompass more than 70 mesenchymal malignancies yet constitute less than 1% of adult cancers [1]; their crude incidence is 3.3 per 100,000 in the United States [2], 3.3–4.7 per 100,000 across Europe [3], and yields roughly 330 new cases annually in Switzerland (https://www.krebsliga.ch/ueber-krebs/krebsarten/weichteilkrebs-weichteilsarkome#:~:text=Weichteilkrebs%20(Weichteilsarkome),knapp%207%25%20aller%20Krebserkrankungen%20aus (accessed on 17 September 2025)) Despite multimodal advances, 5-year overall survival (OS) remains 55–65%, underscoring a persistent mortality burden that is disproportionate to their rarity [4].

Definitive treatment of localized STS rests on limb-sparing surgery plus radiotherapy [5]; systemic chemotherapy is optional in high-risk patients, and its peri-operative utility remains contentious [6]. Because these medicines can cause serious side effects, patients and doctors need reliable numbers to weigh the risks against the limited benefits they usually provide [7]. Proponents argue that anthracycline-based regimens may eradicate occult micro metastases [8], whereas sceptics underscore that no randomized trial has demonstrated a convincing overall survival advantage for localized disease and that, in the metastatic setting, chemotherapy yields no clinically meaningful benefit beyond brief tumour control [9]. When used, doxorubicin—often paired with ifosfamide—remains the reference backbone [10], and newer targeted or immune agents (pazopanib, regorafenib, pembrolizumab) extend progression-free survival by only weeks to months without improving overall survival or patient-reported outcomes [1,8,11]. Given these modest efficacy signals, rigorous quantification of treatment-related early/transient and late/persistent toxicity becomes paramount to inform risk–benefit discussions and trial design [12,13,14].

Systemic therapy is frequently limited by—usually transient and reversible—hematological and gastrointestinal toxicities, which trigger dose reductions, delays, and early discontinuation [15]; reported grade ≥ 3 AE frequencies vary widely because trials employ disparate grading scales, selective event reporting, and inconsistent denominators, complicating efforts to balance these toxicities against the modest efficacy benefits observed.

No up-to-date synthesis rigorously harmonizes AE definitions, normalizes event rates to cohort size, and interrogates reporting quality across both historical and contemporary RCTs, leaving clinicians without reliable toxicity benchmarks [16] and hindering the design of future trials that can meaningfully weigh risk against benefit in this rare cancer.

This systematic review seeks to resolve the uncertainty around chemotherapy-related toxicity in STS by answering a single overarching question: “What is the nature, frequency, and treatment attribution of adverse events in systemic therapy for soft tissue sarcoma, and how consistently are these events reported across randomized controlled trials?” To this end, we will (i) pool and normalize the incidence of CTCAE grade ≥ 3 and grade 4 toxicities across all eligible RCTs; (ii) compare composite toxicity burdens between conventional anthracycline-based regimens and newer targeted or immune therapies; (iii) calculate treatment-versus-control risk differentials where parallel safety data exist; and (iv) critically appraise AE reporting practices to identify methodological deficits and propose harmonized, CTCAE v5.0-driven standards for future sarcoma trials.

## 2. Materials and Methods

This systematic review and meta-analysis was conducted in accordance with PRISMA 2020. The protocol was registered prospectively in PROSPERO (CRD420251087366); any deviations are noted below.

The selection process followed predefined PICO criteria:

Patient/Population: patients with histologically confirmed soft tissue sarcoma (STS) of any subtype or age.
Intervention: systemic therapy (e.g., cytotoxic chemotherapy, kinase inhibitors, or immune checkpoint inhibitors) administered in the adjuvant, neoadjuvant, or palliative setting.
Comparison: no systemic therapy, i.e., surgery ± radiotherapy, best supportive care, or placebo.
Outcome: incidences of grade ≥ 3 adverse events (AEs); secondary outcomes: overall survival and treatment-related mortality.
Study type: randomized controlled trials.

Exclusion criteria were duplicate publications, non-English language, and absence of extractable AE data.

A professional librarian designed comprehensive search strings (Supplementary Table S1). PubMed, CENTRAL, and Google Scholar were queried from inception to 8 January 2025. Searches were updated on 16 April 2025.

**Figure 1:**
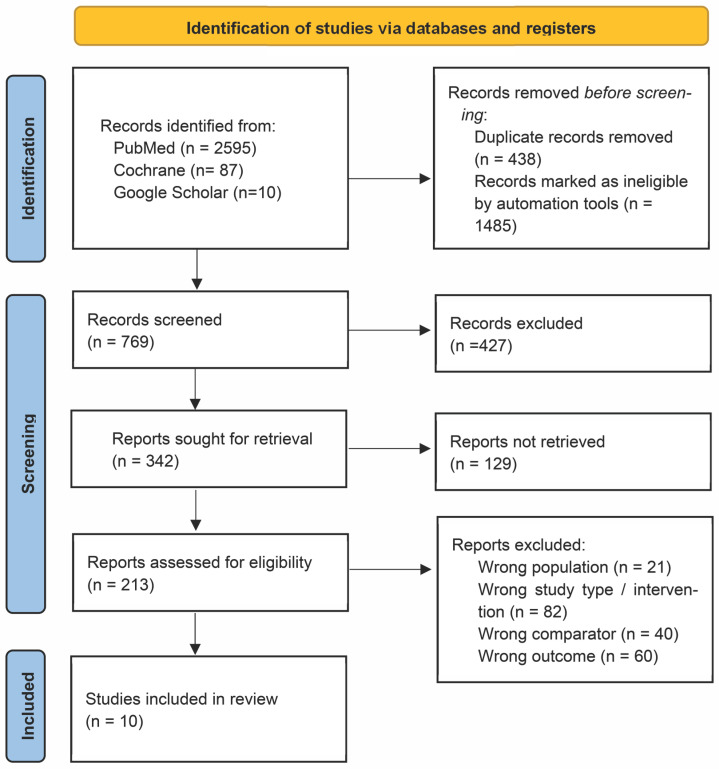
PRISMA 2020 flow diagram.

Two reviewers (RA, PH) independently screened titles and abstracts, assessed full texts, and resolved discrepancies by consensus with a third reviewer (BF). Reasons for exclusion at the full-text stage are detailed in Supplementary Table S2. The selection process is illustrated in a PRISMA 2020 flow diagram (Figure 1).

Using a piloted electronic form, two reviewers extracted study characteristics (author, year, design, setting, drugs, dosing, sample size, follow-up) and outcome data (numerators and denominators for AEs by grade, survival estimates). Disagreements were resolved by a third reviewer. The screening tool Rayyan (Rayyan: AI-Powered Systematic Review Management Platform) was used to screen for duplicates and detection of wrong study types.

Study characteristics are summarized in Table 1.

AE terms reported as WHO grades or narrative descriptors were recoded to CTCAE v5.0 organ classes using a predefined mapping key (https://dctd.cancer.gov/research/ctep-trials/for-sites/adverse-events/ctcae-v5-5x7.pdf (accessed on 17 September 2025)) For each study, we identified the denominator used for toxicity reporting; frequencies were normalized to “patients evaluable for safety”. Trials lacking an explicit denominator were excluded from quantitative synthesis but retained for qualitative comment.

Randomized trials were appraised with the Cochrane RoB 2 tool. Appraisal was performed by one reviewer (RA) and the support of ChatGPT v.4. Detailed judgements are provided in Supplementary Table S3 and Figure S1.

The primary endpoint was the pooled incidence of CTCAE grade ≥ 3 AEs. Further measures included grade 4 AE incidence, normalized AE burden (events per 100 treated patients), and treatment-versus-control risk ratios (RRs) in selected toxicity classes.

Pooled proportions were computed on logit-transformed data using a DerSimonian–Laird random-effects model when ≥3 studies were available; otherwise, results are presented descriptively. Special attention was also given to late-effect toxicities under the supervision of a fourth reviewer (GS). Normalized AE burden was calculated as (events ÷ patients evaluable for safety) × 100. Between-group comparisons (anthracycline vs. kinase inhibitor regimens) employed unpaired two-tailed *t*-tests (*α* = 0.05). Treatment effect RRs were calculated when both arms reported events. Statistical heterogeneity was quantified with *I^2^* and *τ^2^* in the DerSimonian–Laird random-effects model analyses, and 95% CIs were calculated using the Hartung–Knapp method. Analyses were performed in R 4.3.2 using the meta and metafor packages; figures were drafted in Excel 365.

The complete extraction dataset, statistical code, and figure templates are openly available on Zenodo (doi: 10.5281/zenodo.15848465).

Tables S1–S3 (Full search strings), Table S4 (Excluded full texts, with reasons), Table S5 (Detailed judgments), and Figure S1 (Risk of bias plot) accompany this article. The publicly deposited dataset and R scripts enable independent verification of all analyses.

## 3. Results

### 3.1. CTCAE Categories and Denominator Definitions

Eight of the ten eligible randomized trials reported sufficient safety data, providing 978 chemotherapy-exposed patients; three placebo control cohorts contributed 278 patients. Patient numbers per treatment arm ranged widely—from 45 to 239—so later frequency estimates are, if not otherwise specified, expressed with explicit numerators and denominators.

Adverse event terminology varied across time. Earlier trials used the WHO grading system or collapsed events into broad “clinical” versus “laboratory” categories, whereas more recent studies applied CTCAE v4.0 or v5.0. To ensure comparability, all reported events were recoded into the corresponding CTCAE organ classes (Table 2 and Table 3).

Denominator definitions also differed: five trials reported AEs per treated patient, two per intention-to-treat population, and one failed to define its safety cohort. To check for every quantitative analysis that follows, event frequencies are therefore normalized to the number of patients explicitly evaluable for safety in each arm. This approach establishes a transparent foundation for comparing toxicity profiles across systemic therapy regimens in soft tissue sarcoma.

### 3.2. Frequency of CTCAE Grade 3–4 Adverse Events Across Trials

**Figure 2:**
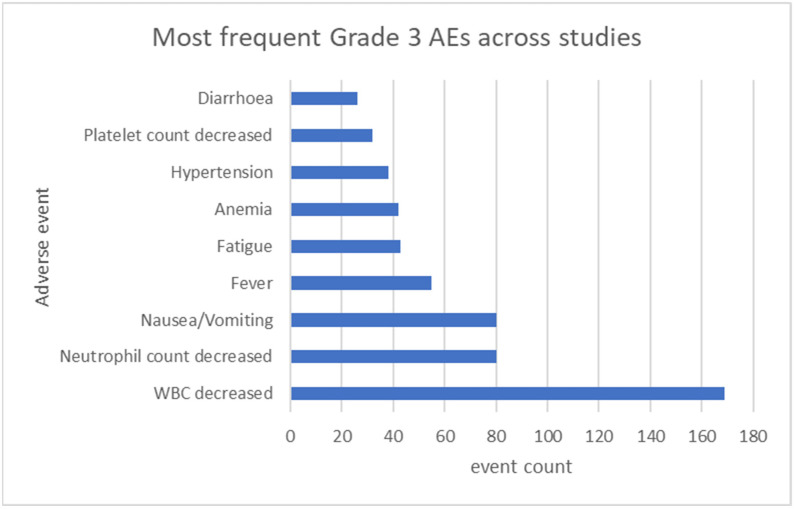
Bar plot of most frequent grade 3 AEs.

Pooled across the eight trials, grade 3 adverse events were documented in 978 treated patients, while grade 4 events were reported separately in seven studies (Table 4 and Table 5). The frequently mentioned AE “alopecia” had to be excluded from all analyses, since it only exists as grade 1 or 2 AE in CTCAE v5.0. Leucopenia emerged as the most common grade 3 toxicity, occurring 169 times in the treated patients overall. Gastrointestinal events—principally nausea/vomiting—followed, at 80 cases, and fever was reported 55 times (Figure 2).

**Figure 3:**
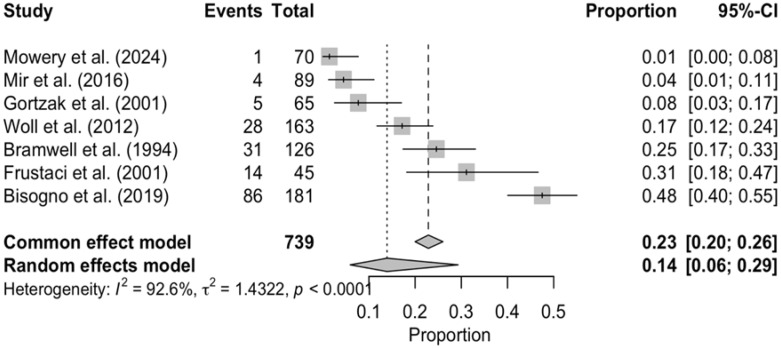
Forest plot of grade 3 leucopenia. This forest plot represents the absolute event count of grade 3 leucopenia in every study that reported this kind of AE, sorted in ascending order of frequency. Pooled effect estimates are denoted by the diamond. Mowery et al. (2024) [19]; Mir et al. (2016) [15]; Gortzak et al. (2001) [20]; Woll et al. (2012) [17]; Bramwell et al. (1994) [21]; Frustaci et al. (2001) [18]; Bisogno et al. (2019) [24].

**Figure 4:**
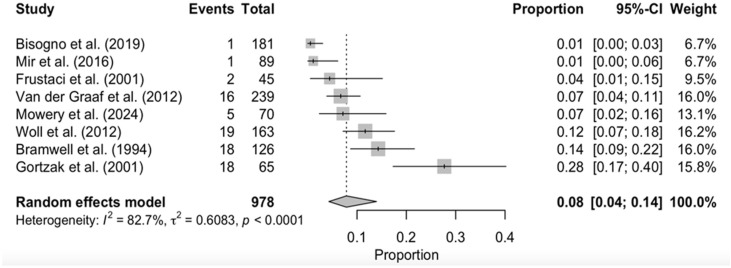
Forest plot of grade 3 nausea/vomiting. This forest plot represents the absolute event count of grade 3 nausea/vomiting in every study that reported this kind of AE, sorted in ascending order of frequency. Pooled effect estimates are denoted by the diamond. Bisogno et al. (2019) [24]; Mir et al. (2016) [15]; Frustaci et al. (2001) [18]; Van der Graaf et al. (2012) [11]; Mowery et al. (2024) [19]; Woll et al. (2012) [17]; Bramwell et al. (1994) [21]; Gortzak et al. (2001) [20].

Subgroup DerSimonian–Laird random-effects model analyses were performed for two selected adverse events, “Leucopenia” and “Nausea/Vomiting” (Figure 3 and Figure 4). Both analyses showed significant between-study heterogeneity (*p* < 0.0001).

### 3.3. Toxicity-Related Deaths and Treatment Discontinuation

Six studies reported toxic deaths, of which there were cumulatively four (0.4% of the treated patients). In total, 71 treatment discontinuations due to adverse events (5.5% of the treated patients) were noted.

### 3.4. Persisting/Late-Term Toxicities

Often persisting, late-term toxicities, such as cardiac, neurological, nephrological, and hepatic toxicities, were rarely assessed/reported as relatively uncommon, occurring in <1% of the patients. Cardiac toxicity, notably grade 3 decreases in left ventricular ejection fraction, were noted in five patients, while grade 3 hepatic toxicity, represented by ALAT elevation, occurred three times. No grade 3 nephrological toxicity (creatinine increase) or grade 3 ASAT elevation was observed. Clinically frequently observed life quality-defining neuropathic side effects were basically not reported.

Only two grade 4 long-lasting AEs were reported.

Compared to acute events, late-effect toxicities were reported rarely.

### 3.5. Normalized Grade 3 Toxicity Burden

**Figure 5:**
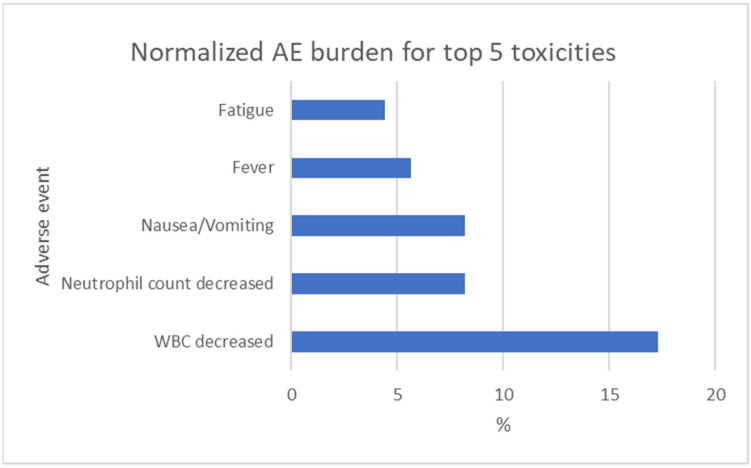
Normalized AE burden for top 5 toxicities.

Because cohort sizes varied markedly, each grade 3 event was re-expressed as “patients affected per 100 treated” (Table 6 and Table 7; Figure 5). After normalization, leucopenia affected, on average, 17 patients per 100 (95% CI 14–20); gastrointestinal toxicities clustered at 8–11 per 100, and fatigue at 4 per 100.

Normalization sharpens inter-study comparisons. For example, 18 cases of nausea/vomiting translated into 28% of patients in the 65-patient Gortzak trial [20] but only 14% in the 126-patient Bramwell study [21].

Grade 4 toxicities were widely distributed and almost exclusively hematologic. Three out of seven studies reported 27 to 45% grade 4 hematologic toxicity and the others < 1%. Non-hematologic grade 4 events were observed in fewer than 12% of patients. Nevertheless, reporting heterogeneity remains substantial: contemporary trials provided detailed and exhaustive matrices, whereas older studies listed only selected events.

**Figure 6:**
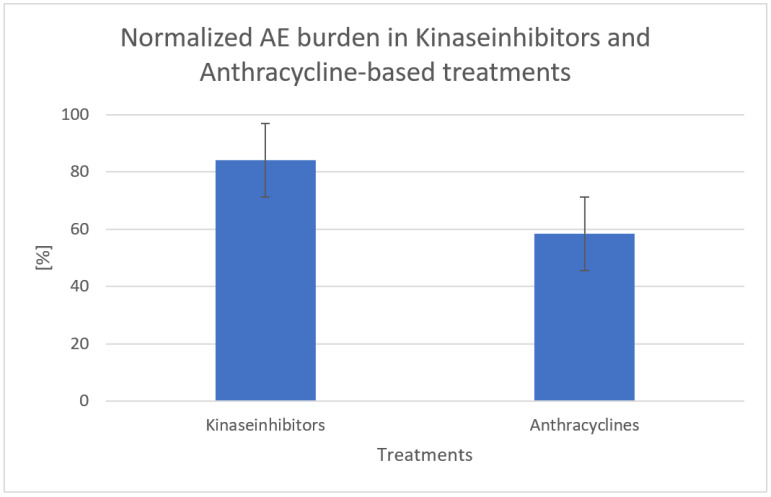
Toxicity burdens pooled by mechanism.

Subgroup analysis of the cumulative normalized toxicity burden (indicated in [%] resulting from the total number of grade 3 AE divided by 100) in kinase-inhibitor-based and anthracycline-based treatments is shown in In Figure 6. The two regimens showed different composite rates but without statistical significance (84% vs. 58%; *p* = 0.64), suggesting that raw count disparities stemmed largely from sample-size differences rather than inherent pharmacological drug choice. The kinase-inhibitor-based treatment class includes the Van der Graaf et al. (Pazopanib) [11] and the Mir et al. (Regorafenib) [15] studies, whereas anthracyclines were used in the Woll [17], Frustaci [18], Bramwell [21], and Gortzak [20] studies. The remaining two studies used a PD-1 antibody (Pembrolizumab) and an alkaloid (Vinorelbine) and were excluded from this subgroup analysis for better comparability.

### 3.6. Treatment-Versus-Control Comparison of Toxicities

Three trials administered placebo to their control group, whereas the remaining protocols compared systemic therapy to surgery or radiotherapy alone. Therefore, only van der Graaf 2012 (pazopanib) [11], Mir 2016 (regorafenib) [15], and Mowery 2024 (pembrolizumab) [19] reported grade 3–4 adverse events (AEs) for both the experimental and control arms, which are listed in Table 8.

Across these studies, the experimental regimens consistently produced higher rates of common grade 3 toxicities. Fatigue rose from 5% to 31% in van der Graaf [11] and from 7% to 13% in Mir [15]; gastrointestinal events (nausea/vomiting, diarrhea) increased by 4–8 percentage points, and hematologic events by 6–12 percentage points. A pooled *t*-test of the study-level differences yielded *p* = 0.001, confirming an overall significant excess of toxicity with active treatment.

**Figure 7:**
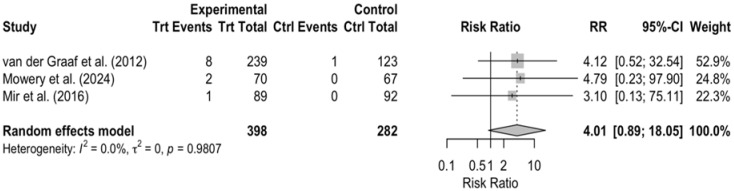
Treatment vs. control comparison—grade 3 vomiting. When analyzing one single AE, there is an indication of an increased risk in the treatment group compared to the control group (RR > 1), but the results remain insignificant, since the CI is wide and including 1. All studies show the same tendencies, resulting in very low between-study heterogeneity *I*^2^ = 0.0%. Pooled effect estimates are denoted by the diamond. Van der Graaf et al. (2012) [11]; Mowery et al. (2024) [19]; Mir et al. (2016) [15].

**Figure 8:**
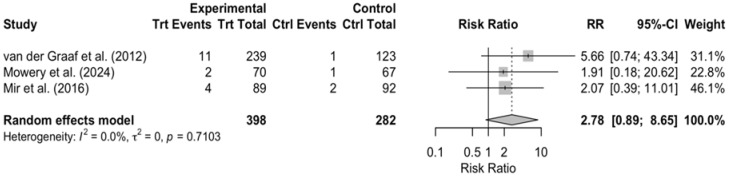
Treatment vs. control comparison—grade 3 diarrhea. When analyzing one single AE, there is an indication of an increased risk in the treatment group compared to the control group (RR > 1), but the results remain insignificant, since the CI is wide and including 1. All studies show the same tendencies, resulting in very low between-study heterogeneity *I*^2^ = 0.0%. Pooled effect estimates are denoted by the diamond. Van der Graaf et al. (2012) [11]; Mowery et al. (2024) [19]; Mir et al. (2016) [15].

On the other hand, subgroup DerSimonian–Laird random-effects model analyses were performed for two selected adverse events, “Vomiting” and “Diarrhea” (Figure 7 and Figure 8), showing only an indication of an increased risk in the treatment group, without statistical significance (RR > 1, 95% CI 0.89–18.5 and 0.89–8.65, respectively).

**Figure 9:**
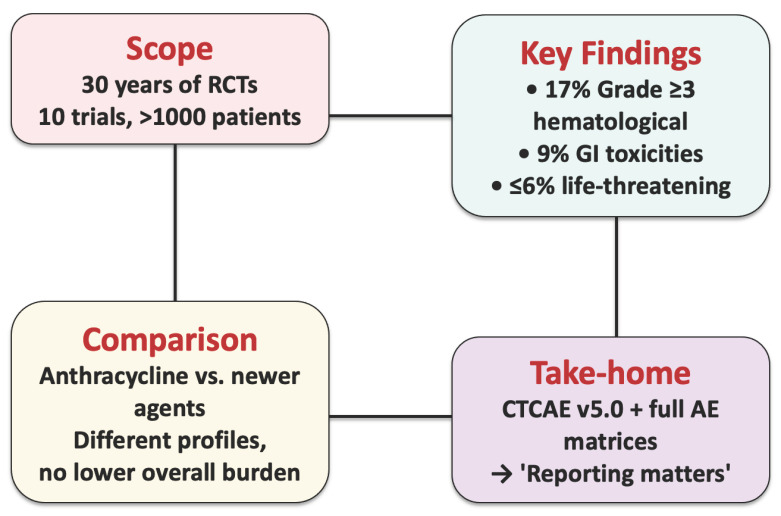
Overview of a 30-year systematic review of randomized STS systemic therapy trials (>1000 patients). Severe adverse events are common (≈17% hematological, ≈9% gastrointestinal, ≤6% life-threatening). Toxicity profiles differ between drug classes, but newer agents are not overall safer. Rigorous CTCAE v5.0 reporting and full AE matrices are required for reliable risk–benefit appraisal.

Interpretation of these findings is limited by incomplete and asymmetric AE reporting in the control arms of several earlier trials, where toxicities were either not collected prospectively or summarized only qualitatively (Figure 9). This reporting imbalance introduces bias and underlines the need for future sarcoma studies to document toxicities consistently.

## 4. Discussion

This systematic review synthesizes three decades of randomized evidence and shows that severe adverse events (AEs) in systemic therapy for soft tissue sarcoma are moderately frequent yet markedly under-reported—mainly regarding potentially persistent late effects. After rigorous CTCAE re-mapping and denominator normalization, grade ≥ 3 hematological toxicities occurred in roughly one in six treated patients, severe gastrointestinal events in one in ten, and grade 4 events in no more than 6%. Despite this apparently manageable profile, substantial gaps in AE capture—two trials with insufficient toxicity data and seven lacking parallel control-arm reporting—limit the reliability of risk estimates and undermine confidence in risk–benefit appraisal.

Pooling 978 chemotherapy-exposed patients across eight RCTs revealed a consistent, “predictable” toxicity hierarchy: leucopenia dominated (17 patients/100), followed by nausea/vomiting (8–11/100) and fatigue (~4/100). Late-effect toxicities (cardiac, hepatic, neurological, and nephrological) were rarely assessed and reported, occurring only in <1% of the patients. Normalized burdens did not differ between anthracycline and kinase-inhibitor regimens (58% vs. 84%; *p* = 0.64), indicating that drug class selection alone is unlikely to mitigate severe toxicity (Figure 6). Where control data were available, experimental therapy increased common grade-3 AEs by 4–12 percentage points (*p* = 0.001), underscoring a tangible but clinically non-negotiable safety cost (Table 8).

Viewed through a bedside lens, the numbers translate into a clinically non-trivial but broadly manageable and predominantly short-term risk profile. A leucopenia rate of 17% (≈1 in 6 patients) is comparable to that seen with modern anthracycline regimens in breast cancer, where grade ≥ 3 neutropenia ranges from 20 to 30%—yet the efficacy dividend in STS is far smaller. In PALETTE (pazopanib) [11], the 3-month PFS gain was accompanied by a 14% AE-related discontinuation rate; REGOSARC (regorafenib) [15] improved median PFS by 1.6 months while prompting dose reductions or delays in 48% of patients. Across the eight trials with safety data, discontinuations for toxicity clustered between 1% and 14% (on average 5.5%), indicating that severe AEs routinely truncate therapy and may nullify any marginal survival benefit. Accordingly, chemotherapy in localized, high-risk STS remains discretionary: clinicians must weigh a roughly 10–20% chance of therapy-limiting toxicity against PFS benefits measured in weeks and tailor prophylactic measures (G-CSF and guideline-based anti-emetic triplets) and close monitoring for the subset of patients who opt for systemic treatment.

Our toxicity estimates diverge notably from earlier sarcoma reviews, which cited grade ≥ 3 hematological rates as high as 40% and gastrointestinal rates exceeding 20% [25,26]. Those analyses pooled absolute counts from single-arm phase II studies or mixed heterogeneous denominators, thereby inflating frequencies in trials with small cohorts. After harmonizing denominators to “patients evaluable for safety,” our leucopenia figure fell by ~50% and severe nausea/vomiting by ~60%, showing that denominator choice, rather than pharmacologic progress, explains much of the perceived decline. A second driver is the evolution of supportive care: nowadays, routinely administered prophylactic G-CSF, 5-HT_3_ antagonists and NK_1_ blockers, largely absent in trials from the 1990s, result in moderate myelosuppression and emesis. Consequently, our findings refine—not refute—earlier warnings by demonstrating that severe toxicity remains common but is quantitatively lower once methodological artefacts are removed and contemporary supportive measures are considered.

Methodological scrutiny reveals that reporting practices, not pharmacology, are the chief obstacle to reliable toxicity estimation. Of the ten eligible RCTs, seven failed to publish parallel control-arm AE matrices and two limited toxicity data to a very low level, forcing their exclusion from quantitative synthesis and inflating imprecision. Legacy WHO grading or narrative terms persisted in four trials, necessitating post hoc CTCAE mapping and introducing potential misclassification (Table 1). Denominators were equally problematic: only five studies specified a “patients-evaluable-for-safety” cohort, while two defaulted to intention-to-treat counts and one left the base population undefined. These deficits translated into serious or very serious concerns in the “Outcome Measurement” domain of our RoB 2 assessment for 4/10 trials (Supplementary Table S3). Encouragingly, all trials published since 2015 adopted CTCAE v4.0/5.0 and provided grade-stratified counts, indicating a shift toward better practice; however, until explicit denominators and full AE matrices become mandatory, certainty in toxicity estimates—and thus informed consent—will remain low. Our findings are supported by the results of a similar study, published in 2019 already, and also call for reporting to be carried out with more scrutiny [27].

The present study offers the largest pooled safety dataset in STS to date, achieved through an exhaustive multi-database search, duplicate screening, double data extraction, and public release of the full dataset and R code—steps that bolster transparency and reproducibility. A further strength is the systematic harmonization of AE terminology to CTCAE v5.0, which enabled denominator-normalized comparisons across three decades of trials and reduced inflation artefacts seen in prior reviews. Nevertheless, several constraints temper our conclusions. First, the evidence base is numerically small (ten RCTs; 1026 treated patients), with two trials providing insufficient toxicity data and seven lacking control-arm matrices. Second, between-study heterogeneity remained substantial for gastrointestinal and hematological AEs (*I*^2^ > 60%, Figure 3 and Figure 4), and publication bias could not be assessed with so few studies.

This pooled data furnishes pragmatic toxicity benchmarks for patient counselling and supportive care planning. When systemic therapy is contemplated—typically as an optional adjunct in high-risk localized disease or for palliation—[28] clinicians can quote a 17% risk of grade ≥ 3 myelosuppression and an 8–11% risk of severe gastrointestinal events, with life-threatening toxicities seldom exceeding 6% (Table 7). Prophylactic G-CSF should therefore be standard when the anticipated incidence of grade ≥ 3 neutropenia exceeds 20% [29], and modern anti-emetic triplets (5-HT_3_, NK_1_, dexamethasone [30]) are justified even for “moderately emetogenic” protocols. Because toxicity-driven discontinuation reached up to 14% in the reviewed trials, early integration of dose modification algorithms and real-time toxicity monitoring is essential. Most importantly, these figures should be presented as provisional lower bounds—acknowledging reporting gaps—in shared decision-making, enabling patients to weigh a tangible toxicity risk against often modest gains in progression-free or overall survival.

To convert “moderate and uncertain” toxicity estimates into high-certainty risk–benefit data, future sarcoma trials must treat adverse event capture as a primary endpoint rather than an afterthought. Reporting should follow a CTCAE v5.0 template with explicit safety denominators in both study arms, full grade stratification, and adherence to CONSORT-Harms extensions; the complete AE matrix should accompany every publication as a searchable supplement or within open repositories. Prospective safety pooling consortia—such as an expanded EORTC STS Net—could aggregate uniform toxicity data across simultaneously running studies, rapidly surpassing the 1000-patient threshold needed for precise, subtype-specific estimates. Regulators and funding bodies should incentivize such transparency by linking trial approval or reimbursement to publicly accessible AE datasets and by mandating inclusion of patient-reported outcome measures that capture symptomatic burdens often missed in clinician-graded CTCAE logs [31]. Only through these procedural guardrails can forthcoming systemic-therapy trials deliver toxicity evidence commensurate with their efficacy claims.

## 5. Conclusions

Systemic therapy for soft tissue sarcoma yields a moderate yet clinically relevant burden of severe acute adverse events—about one in six patients develops grade ≥ 3 myelosuppression and one in ten a severe gastrointestinal toxicity—while truly life-threatening events remain of ≤6%. However, the certainty of these estimates is constrained by inconsistent denominators, legacy grading scales, and missing control-arm data. Potentially persisting late-term effects were found to be substantially under-reported. “Reporting matters”: without harmonized, CTCAE v5.0-based toxicity capture and open publication of full AE matrices, risk–benefit appraisals will continue to rest on shaky ground. Future sarcoma trials must elevate rigorous AE reporting to the same priority as efficacy if patients and clinicians are to make informed, evidence-based treatment decisions.

## Figures and Tables

**Table 1:** Study characteristics.

Study Title or Registration Number	Trial Type	Setting	Chemotherapy	Dose or Schedule	Patient Count (Treatment/Control)	Median Follow-Up, Months	Evaluated Outcomes
Pazopanib for metastatic soft-tissue sarcoma (PALETTE) [11]	Randomized, double-blind, placebo-controlled phase 3 trial	Adjuvant	Pazopanib		246/123	14.9	PFS, OS, response rate, safety and quality of life
EORTC 62931 [17]	Multicenter randomized controlled trial	Adjuvant	Doxorubicin, Ifosfamide, Lenograstim	800 mg daily	175/176	95.88	PFS, OS, toxic effects
Adjuvant chemotherapy for adult soft tissue sarcomas of the extremities and girdles [18]	Randomized cooperative trial	Adjuvant	Epidoxorubicin, Ifosfamide	Five cycles of doxorubicin 75 mg/m^2^, ifosfamide 5 g/m^2^, and lenograstim every 3 weeks	53/51	59	DFS, OS, toxicity
SU2C-SARC032 [19]	Randomized clinical trial	Neoadjuvant	Pembrolizumab	5 cycles of 4′-epidoxorubicin 60 mg/m^2^ days 1 and 2 and ifosfamide 1.8 g/m^2^ days 1 through 5, with hydration, mesna, and granulocyte colony-stimulating factor	64/63	43	DFS, OS, grade 3 or higher adverse events
Neo-adjuvant chemotherapy for ‘high-risk’ adult soft tissue sarcoma [20]	Randomized phase II study	Neoadjuvant	Doxorubicin and Ifosfamide	200 mg i.v. every 3 weeks	67/67	88	Estimated 5-year DFS, 5-year OS, toxicity
Adjuvant CYVADIC chemotherapy for adult soft tissue sarcoma [21]	Randomized controlled trial	Adjuvant	Cyclophosphamide, Vincristine, Doxorubicin, Dacarbazine	3 cycles of 3-weekly doxorubicin 50 mg/m^2^ intravenous (i.v.) bolus and ifosfamide 5 g/m^2^ (24 h infusion)	145/172	80	RFS, OS, local recurrence, distant metastases, toxicity
Intensified adjuvant IFADIC chemotherapy in combination with radiotherapy versus radiotherapy alone for soft tissue sarcoma: long-term follow-up of a prospective randomized feasibility trial [22]	Randomized controlled trial	Adjuvant	Ifosfamide, Dacarbazin, Doxorubicin	Ifosfamide (1500 mg/m^2^, days 1–4), DTIC (200 mg/m^2^, days 1–4) and doxorubicin (25 mg/m^2^, days 1 and 2) i.v., in a 14-day cycle.	31/28	97	RFS, time to local failure, time to distant failure, OS
Eastern Cooperative Oncology Group: a comparison of adjuvant doxorubicin and observation for patients with localized soft tissue sarcoma [23]	Randomized controlled trial	Adjuvant	Doxorubicin	70 mg/m^2^ (slow push, every 3 weeks for seven courses for a maximum of 550 mg/m^2^)	17/13	30	DFS, OS
Vinorelbine and continuous low-dose cyclophosphamide in patients with high-risk rhabdomyosarcoma [24]	Multicenter, open-label, randomized, phase 3 trial	Maintenance Therapy	Vinorelbine, Cyclophosphamide	Cyclophosphamide 500 mg/m^2^ intravenously (i.v.) bolus on day 1, vincristine 1.4 mg/m^2^ i.v. bolus on day 1, doxorubicin (Adriamycin; Adria Laboratories, Columbus, OH, USA) 50 mg/m^2^ i.v. bolus on day 1, and dacarbazine (DTIC) 400 mg/m^2^ by 1-h infusion on days 1 to 3 (CYVADIC) cycles repeated every 28 days for eight courses	185/186	60.3	5-year DFS, 5-year OS, toxicity
Safety and efficacy pf regorafenib in patients with advanced soft tissue sarcoma [15]	Randomized, double-blind, placebo-controlled, phase 2 trial		Regorafenib	6 cycles of i.v. vinorelbine 25 mg/m^2^ on days 1, 8, and 15, and daily oral cyclophosphamide 25 mg/m^2^, on days 1–28	90/92	20	PFS, grade 3 or higher adverse events

**Table 2:** CTCAE categories and denominators for toxicity reporting.

Adverse Event	Van der Graaf et al. (2012) [11]	Woll et al. (2012) [17]	Frustaci et al. (2001) [18]	Mowery et al. (2024) [19]
General disorders [%]	13	1	0	6
Fatigue	30	nr	0	1
Fever	nr	1	0	0
Pain	nr	nr	0	3
Gastrointestinal disorders [%]	13	15	16	10
Diarrhea	11	0	0	2
Nausea/vomiting	16	19	2	5
Oral mucositis	3	5	5	0
Investigations [%]	0	43	38	1
Weight loss	0	nr	0	0
Increased creatinine	nr	0	0	0
Increased Alanine aminotransferase	nr	2	0	0
Increased aspartate aminotransferase	nr	0	0	0
Increased blood bilirubin	nr	1	0	nr
Decreased white blood cell count	nr	28	14	1
Decreased neutrophil count	nr	13	nr	nr
Decreased platelet count	nr	19	3	nr
Vascular disorders [%]	7	nr	0	9
Hypertension	16	nr	0	6
Blood and lymphatic system disorders [%]	nr	7	17	10
Anemia	nr	11	7	7
Infections and infestations [%]	nr	7	0	13
Wound infection	nr	0	0	7
Infection, general	nr	12	0	2
Metabolism and nutrition disorders [%]	6	nr	0	0
Anorexia	14	nr	0	0
Dysgeusia	0	nr	0	0
Skin and subcutaneous tissue disorders [%]	1	nr	0	3
Rash/desquamation	1	nr	0	2
Nervous system disorders [%]	nr	1	0	0
Cardiac disorders [%]	1	1	0	0

**Table 3:** CTCAE categories and denominators for toxicity reporting.

Adverse Event	Gortzak et al. (2001) [20]	Bramwell et al. (1994) [21]	Bisogno et al. (2019) [24]	Mir et al. (2016) [15]
General disorders [%]	nr	nr	29	22
Fatigue	nr	nr	nr	12
Fever	nr	nr	53	1
Pain	nr	nr	nr	7
Gastrointestinal disorders [%]	28	14	6	10
Diarrhea	nr	nr	9	4
Nausea/vomiting	18	18	1	1
Oral mucositis	nr	nr	nr	4
Investigations [%]	8	31	85	8
Weight loss	nr	nr	nr	nr
Increased creatinine	0	nr	nr	0
Increased Alanine aminotransferase	nr	0	nr	nr
Increased aspartate aminotransferase	nr	0	nr	1
Increased blood bilirubin	nr	0	nr	nr
Decreased white blood cell count	5	31	86	4
Decreased neutrophil count	nr	nr	66	1
Decreased platelet count	0	8	1	1
Vascular disorders [%]	nr	nr	nr	18
Hypertension	nr	nr	nr	16
Blood and lymphatic system disorders [%]	nr	nr	9	3
Anemia	nr	nr	16	3
Infections and infestations [%]	0	2	2	3
Wound infection	0	nr	nr	nr
Infection, general	0	3	3	3
Metabolism and nutrition disorders [%]	nr	5	nr	3
Anorexia	nr	6	nr	3
Dysgeusia	nr	nr	nr	nr
Skin and subcutaneous tissue disorders [%]	nr	nr	1	4
Rash/desquamation	nr	nr	1	4
Nervous system disorders [%]	1	5	1	1
Cardiac disorders [%]	1	2	0	1

In Table 2 and Table 3, a reasonable range of adverse events reported across the studies is summarized. Not every subtype of adverse event was reported in every study. The concerned cells are annotated with “nr”= not reported (e.g., “fever” in Van der Graaf et al. [11]). We classified the adverse events in the CTCAE categories, which are highlighted in blue. The most frequently reported clinical and laboratory adverse events overall are highlighted in salmon. It is notable that the adverse events themselves are listed in absolute numbers, whereas the classifications are frequencies (%), which means that in the Woll et al. study [17], about 15% of the treated patients had a gastrointestinal adverse event—11 cases of diarrhea and 16 and 3 of nausea/vomiting and oral mucositis, respectively. One patient could also have more than one AE, since the protocols did not give information about which patients suffered from what kind of adverse event.

**Table 4:** Absolute event count of grade 3 adverse events across studies.

Adverse Event	Van der Graaf et al. (2012) [11]	Woll et al. (2012) [17]	Frustaci et al. (2001) [18]	Mowery et al. (2024) [19]	Gortzak et al. (2001) [20]	Bramwell et al. (1994) [21]	Bisogno et al. (2019) [24]	Mir et al. (2016) [15]
Fatigue	30	nr	0	1	nr	nr	nr	nr
Diarrhea	11	0	0	2	nr	nr	9	4
Nausea/vomiting	16	19	2	5	18	18	1	1
Weight loss	0	nr	0	0	nr	nr	nr	nr
Hypertension	16	nr	0	6	nr	nr	nr	16
Anorexia	14	nr	0	0	nr	6	nr	3
Dysgeusia	0	nr	0	0	nr	nr	nr	nr
Rash/desquamation	1	nr	0	2	nr	nr	1	nr
Mucositis	1	5	5	0	nr	nr	nr	4
Fever	nr	1	0	0	nr	nr	53	1
(Skin) infection	nr	12	0	2	0	3	3	3
Neurological problem	nr	1	0	0	1	5	5	nr
Cardiac	16	1	0	0	1	2	0	1
Pain	nr	nr	0	3	nr	nr	nr	7
Wound infection	nr	nr	0	7	nr	nr	nr	nr
Creatinine	nr	0	0	0	0	nr	nr	nr
Bilirubin	nr	1	0	nr	nr	0	nr	0
AST elevated	nr	0	0	0	nr	0	nr	1
ALT elevated	nr	2	0	0	nr	0	nr	1
Leucopenia	nr	28	14	1	5	31	86	4
Neutropenia	nr	13	nr	nr	nr	nr	66	1
Thrombocytopenia	nr	19	3	nr	0	8	1	1
Anemia	nr	11	5	7	nr	nr	16	3

This table shows the absolute event counts of the most frequently reported CTCAE grade 3 over all eight studies eligible for this analysis. The sum of the events does not represent the number of patients who had a grade 3 adverse event, since one patient could have had more than one side effect and the studies did not provide us with clear patient profiles. Not every subtype of adverse event was reported in every study. The concerned cells are annotated with “nr”.

**Table 5:** Absolute event count of grade 4 adverse events across studies.

Adverse Event	Van der Graaf et al. (2012) [11]	Woll et al. (2012) [17]	Frustaci et al. (2001) [18]	Mowery et al. (2024) [19]	Gortzak et al. (2001) [20]	Bramwell et al. (1994) [21]	Bisogno et al. (2019) [24]	Mir et al. (2016) [15]
Fatigue	1	nr	0	0	nr	nr	nr	0
Diarrhea	0	0	0	0	nr	nr	0	0
Nausea/vomiting	0	0	0	0	1	1	0	0
Weight loss	0	nr	0	0	nr	nr	nr	nr
Hypertension	0	nr	0	0	nr	nr	nr	1
Anorexia	0	nr	0	0	nr	2	nr	0
Dysgeusia	0	nr	0	0	nr	nr	nr	nr
Rash/desquamation	0	nr	0	0	nr	nr	0	0
Mucositis	0	0	0	0	nr	nr	nr	0
Fever	nr	0	0	1	nr	nr	0	0
(Skin) infection	nr	0	0	0	1	1	0	0
Neurological problem	nr	0	0	0	1	1	1	nr
Cardiac	nr	0	0	0	1	0	0	1
Pain	nr	nr	0	0	nr	nr	nr	0
Wound infection	nr	nr	0	0	nr	nr	nr	0
Creatinine	nr	0	0	0	0	nr	nr	0
Bilirubin	nr	0	0	nr	nr	0	nr	nr
AST elevated	nr	0	0	0	nr	0	nr	0
ALT elevated	nr	0	0	0	nr	0	nr	nr
Leucopenia	nr	41	12	1	0	0	50	0
Neutropenia	nr	44	nr	nr	nr	nr	82	0
Thrombocytopenia	nr	11	2	nr	0	0	1	nr
Anemia	nr	2	1	0	nr	nr	3	1

This table shows the absolute event counts of the most frequently reported CTCAE grade 4 over all eight studies eligible for this analysis. The sum of the events does not represent the number of patients who had a grade 3 adverse event, since one patient could have had more than one side effect and the studies did not provide us with clear patient profiles. Not every subtype of adverse event was reported in every study. The concerned cells are annotated with “nr”.

**Table 6:** Normalized grade 3 AE burden by study size.

Adverse Event	Van der Graaf et al. (2012) [11]	Woll et al. (2012) [17]	Frustaci et al. (2001) [18]	Mowery et al. (2024) [19]	Gortzak et al. (2001) [20]	Bramwell et al. (1994) [21]	Bisogno et al. (2019) [24]	Mir et al. (2016) [15]
Fatigue	13	nr	0	1	nr	nr	nr	13
Diarrhea	5	0	0	3	nr	nr	5	4
Nausea/vomiting	6	12	3	7	28	14	1	1
Weight loss	0	nr	0	0	nr	nr	nr	nr
Hypertension	7	nr	0	9	nr	nr	nr	18
Anorexia	6	nr	0	0	nr	5	nr	3
Dysgeusia	0	nr	0	0	nr	nr	nr	nr
Rash/desquamation	1	nr	0	8	nr	nr	1	nr
Mucositis	1	3	10	0	nr	nr	nr	4
Fever	nr	1	0	0	nr	nr	29	1
(Skin) infection	nr	7	0	7	0	2	31	3
Neurological problem	nr	1	0	0	1	5	1	nr
Cardiac	1	1	0	0	1	2	0	1
Pain	nr	nr	0	4	nr	nr	nr	8
Wound infection	nr	nr	0	10	nr	nr	nr	nr
Creatinine	nr	0	0	0	0	nr	nr	nr
Bilirubin	nr	1	0	nr	nr	0	nr	0
AST elevated	nr	0	0	0	nr	0	nr	0
ALT elevated	nr	2	0	0	nr	0	nr	1
Leucopenia	nr	19	30	1	8	25	48	4
Neutropenia	nr	9	nr	nr	nr	nr	37	1
Thrombocytopenia	nr	13	7	nr	0	6	1	1
Anemia	nr	8	17	10	nr	nr	9	3

This table shows the frequencies of the most frequently reported CTCAE grade 3 over all eight studies eligible for this analysis. Not every subtype of adverse event was reported in every study. The concerned cells are annotated with “nr” = not reported.

**Table 7:** Normalized grade 4 AE burden by study size.

Adverse Event	Van der Graaf et al. (2012) [11]	Woll et al. (2012) [17]	Frustaci et al. (2001) [18]	Mowery et al. (2024) [19]	Gortzak et al. (2001) [20]	Bramwell et al. (1994) [21]	Bisogno et al. (2019) [24]	Mir et al. (2016) [15]
Fatigue	1	nr	0	0	nr	nr	nr	0
Diarrhea	0	0	0	0	nr	nr	0	0
Nausea/vomiting	0	0	0	0	1	12	0	0
Weight loss	0	nr	0	0	nr	nr	nr	nr
Hypertension	0	nr	0	0	nr	nr	nr	1
Anorexia	0	nr	0	0	nr	2	nr	0
Dysgeusia	0	nr	0	0	nr	nr	nr	nr
Rash/desquamation	0	nr	0	0	nr	nr	0	0
Mucositis	0	0	0	0	nr	nr	nr	0
Fever	nr	0	0	1	nr	nr	0	0
(Skin) infection	nr	0	0	0	1	1	0	0
Neurological problem	nr	0	0	0	1	4	1	nr
Cardiac	nr	0	0	0	1	0	0	1
Pain	nr	nr	0	0	nr	nr	nr	0
Wound infection	nr	nr	0	0	nr	nr	nr	0
Creatinine	nr	0	0	0	0	nr	nr	0
Bilirubin	nr	0	0	nr	nr	0	nr	nr
AST elevated	nr	0	0	0	nr	0	nr	0
ALT elevated	nr	0	0	0	nr	0	nr	nr
Leucopenia	nr	28	27	1	0	0	28	0
Neutropenia	nr	30	nr	nr	nr	nr	45	0
Thrombocytopenia	nr	8	7	nr	0	0	1	nr
Anemia	nr	1	7	0	nr	nr	2	1

This table shows the frequencies of the most frequently reported CTCAE grade 4 over all eight studies eligible for this analysis. Not every subtype of adverse event was reported in every study. The concerned cells are annotated with “nr” = not reported.

**Table 8:** Treatment vs. control comparison of grade 3 toxicities.

Adverse Event	Van der Graaf et al. (2012) [11] Treatment Group	Control Group	Mowery et al. (2024) [19] Treatment Group	Control Group	Mir et al. (2016) [15] Treatment Group	Control Group
Fatigue	30	6	1	0	12	6
Diarrhea	11	1	2	1	4	2
Nausea/vomiting	16	3	5	0	1	0
Weight loss	0	0	0	0	nr	0
Hypertension	16	4	6	3	16	2
Anorexia	14	0	0	0	3	4
Dysgeusia	0	0	0	0	nr	nr
Rash/desquamation	1	0	6	4	nr	nr
Mucositis	1	0	0	0	4	0
Fever	nr	nr	0	0	1	1
(Skin) infection	nr	nr	2	0	3	0
Neurological problem	nr	nr	0	0	nr	nr
Cardiac	16	3	0	0	1	0
Pain	nr	nr	7	0	7	5
Wound infection	nr	nr	7	6	nr	nr
Creatinine	nr	nr	0	0	0	0
Bilirubin	nr	nr	nr	nr	nr	nr
Increased AST	nr	nr	0	0	1	1
Increased ALT	nr	nr	0	0	1	1
Leucopenia	nr	nr	1	1	4	5
Neutropenia	nr	nr	nr	nr	1	0
Thrombocytopenia	nr	nr	nr	nr	1	0
Anemia	nr	nr	10	3	3	1

Not every subtype of adverse event was reported in every study. The concerned cells are annotated with “nr” = not reported.

## Data Availability

The complete extraction dataset, statistical code, and figure templates are openly available on Zenodo (doi: 10.5281/zenodo.15848465).

## Supplementary Materials

The following supporting information can be downloaded at https://www.mdpi.com/article/10.3390/cancers17193118/s1, Table S1: PubMed search strategy for clinical studies of patients of all ages with STS (17 December 2024); Table S2: Cochrane search strategy for randomized controlled trials of patients of all ages with STS (16 December 2024); Table S3: Google Scholar search strategy for randomized controlled trials of patients of all ages with STS (8 January 2025); Table S4: Reasons for full-text exclusion [22,23,26,32,33,34,35,36,37,38,39]; Table S5: Detailed judgments [11,15,17,18,19,20,21,22,23,24]; Figure S1: Risk of bias plot.

## Abbreviations

The following abbreviations are used in this manuscript:

STS	Soft tissue sarcoma
RCT	Randomized controlled trial
AE	Adverse event
CTCAE	Common terminology criteria for adverse events
MFS	Metastatic free survival
PFS	Progression free survival
OS	Overall survival
HR	Hazard ratio
