# Supplementary material for: Reporting Matters: Severe Adverse Events in Soft Tissue Sarcoma Therapy—A 30-Year Systematic Review of Placebo- and Non-Systemic-Controlled Randomized Trials
Source: Cancers (Basel). 2025 Sep 25;17(19):3118. doi: 10.3390/cancers17193118 (PMC12524033; doi:10.3390/cancers17193118)
Supplement: Supplementary file 1 [file cancers-17-03118-s001.zip › cancers-3852851-supplementary.pdf]

Supplementary Tables

Supplementary Table S1

PubMed search strategy for clinical studies of patients of all age with STS (soft tissue sarcomas) (17.12.2024)

Search number	Terms	Number of records
	Disease	
#1	"neoplasms, connective and soft tissue"[MeSH Terms] OR sarcoma<br>(which includes:<br>Adenosarcoma<br>Carcinosarcoma<br>Carcinoma 256, Walker<br>Chondrosarcoma<br>Chondrosarcoma, Clear Cell<br>Chondrosarcoma, Mesenchymal<br>Desmoplastic Small Round Cell Tumor<br>Endometrial Stromal Tumors<br>Sarcoma, Endometrial Stromal<br>Fibrosarcoma<br>Dermatofibrosarcoma<br>Neurofibrosarcoma<br>Hemangiosarcoma<br>Histiocytoma, Malignant Fibrous<br>Leiomyosarcoma<br>Liposarcoma<br>Liposarcoma, Myxoid<br>Lymphangiosarcoma<br>Mixed Tumor, Mesodermal<br>Myosarcoma<br>Rhabdomyosarcoma +<br>Myxosarcoma<br>Osteosarcoma<br>Osteosarcoma, Juxtacortical<br>Sarcoma, Ewing<br>Phyllodes Tumor<br>Sarcoma, Alveolar Soft Part<br>Sarcoma, Clear Cell<br>Sarcoma, Experimental<br>Sarcoma 180<br>Sarcoma 37<br>Sarcoma, Avian<br>Sarcoma, Yoshida<br>Sarcoma, Kaposi<br>Sarcoma, Myeloid<br>Sarcoma, Small Cell	318,113 results

	Sarcoma, Synovial)	
	Population	
#2	"Age Groups"[MeSH Terms]<br>(which includes:<br>Adolescent<br>Adult<br>Aged +<br>Middle Aged<br>Young Adult<br>Birth Cohort<br>Child<br>Child, Preschool<br>Infant<br>Infant, Newborn +	10,506,749 results
	Study type	
#3	"Clinical Study"[Publication Type]	1,201,300 results
	Drugs of interest	
#4	"Antineoplastic Agents"[Pharmacological Action] OR "chemotherapy, adjuvant"[MeSH Terms:noexp] OR "Immunotherapy"[MeSH Terms:noexp] OR "Radioimmunotherapy"[MeSH Terms])	1,401,483 results

Supplementary Table S2

Cochrane search strategy for randomized controlled trials of patients of all age with STS (soft tissue sarcomas) (16.12.2024)

Search number	Terms	Number of records
	Disease	
#1	MeSH descriptor: [Sarcoma] explode all trees	1605
	Population	
#2	MeSH descriptor: [Age Groups] explode all trees	720462
	Drugs of interest	
#3	MeSH descriptor: [Chemotherapy, Adjuvant] explode all trees	6108
	Drugs of interest	
#4	MeSH descriptor: [Immunotherapy] explode all trees	12407
#5	#3 OR #4	18423

Supplementary Table S3

Google Scholar search strategy for randomized controlled trials of patients of all age with STS (08.01.2025)

Terms	Number of records
randomized controlled trial, chemotherapy, soft tissue sarcoma	22

Supplementary Table S4

Article	Reason(s) for full text exclusion
EORTC-62092: STRASS[1]	Wrong intervention
Doxorubicin alone versus intensified doxorubicin plus ifosfamide for fi rst-line treatment of advanced or metastatic soft-tissue sarcoma: a randomised controlled phase 3 trial[2]	Wrong comparator
EORTC 62961-ESHO 95 Randomized Clinical Trial[3]	Wrong comparator
Doxorubicin Plus Dacarbazine Versus Doxorubicin Plus Ifosfamide in Combination With Regional Hyperthermia in Patients With Advanced Leiomyosarcoma: A Propensity Score-Matched Analysis[4]	Wrong comparator, wrong outcome
Adjuvant chemotherapy with doxorubicin in high-grade soft tissue sarcoma: a randomized trial of the Scandinavian Sarcoma Group[5]	Wrong comparator, wrong outcome, wrong study design
Neoadjuvant Chemotherapy in High-Risk Soft Tissue Sarcomas: Final Results of a Randomized Trial From Italin (ISG), Spanish (GEIS), French (FSG), and Polis (PSG) Sarcoma Groups[6]	Wrong outcome
Role of adjuvant chemotherapy in the treatment of surgically resected pediatric nonrhabdomyosarcomatous soft tissue sarcomas: A Pediatric Oncology Group Study[7]	Wrong study design, wrong outcome
Outcomes After Preoperative Chemoradiation With or Without Pazopanib in Non-Rhabdomyosarcoma Soft Tissue Sarcoma: A Report From Children's Oncology Group and NRG Oncology[8]	Wrong outcome
A randomized prospective trial using postoperative adjuvant chemotherapy (Adriamycin) in high grade extremity soft-tissue sarcoma[9]	Wrong study design, wrong outcome
Intensified adjuvant IFADIC chemotherapy in combination with radiotherapy versus radiotherapy alone for soft tissue sarcoma: long-term follow-up of a prospective randomized feasibility trial[10]	Wrong outcome
Eastern Cooperative Oncology Group: a comparison of adjuvant doxorubicin and observation for patients with localized soft tissue sarcoma.[11]	Wrong outcome

Supplementary Table S5
Detailed judgments

1. Pazopanib for metastatic soft-tissue sarcoma (PALETTE): a randomised, double-blind, placebo-controlled phase 3 trial [12]
Study Design: Randomized, double-blind, placebo-controlled phase 3 trial.
Strengths:
- Methodology: Double-blind and placebo-controlled → reduced risk of bias
- Multinational and multicenter → higher external validity
- Stratification by histological subtype: Improves interpretability for clinical subgroups.

Weaknesses:
- No significant OS benefit
- Treatment toxicity: Low grade adverse events were relatively common (e.g., hypertension, fatigue, diarrhea).
- Lack of crossover design: Could limit understanding of long-term treatment impact.

2. Adjuvant chemotherapy with doxorubicin, ifosfamide, and lenograstim for resected soft-tissue sarcoma (EORTC 62931): a multicentre randomised controlled trial [13]
Study Design: Multicenter, randomized controlled trial
Strengths:
- Large sample size and multinational scope: Enhances power and generalizability.
- Broad AE reporting
- Rigorous design: Good internal validity due to randomization and standardization.

Weaknesses:
- No significant improvement in either PFS or OS
- Treatment-related toxicity: Chemotherapy arm experienced considerable toxicity (notably hematological).
- Broad inclusion criteria: May dilute benefit in high-risk subgroups.

3. Adjuvant Chemotherapy for Adult Soft Tissue Sarcomas of the Extremities and Girdles: Results of the Italian Randomized Cooperative Trial[14]
Study Design: Randomized, controlled trial focused on high-grade, large (>5 cm) extremity and girdle sarcomas.
Strengths:
- Strict inclusion criteria: Focused on high-risk patients with grade 3–4, large or recurrent tumors → high internal validity
- Statistically significant improvement in PFS and OS.

Weaknesses:
- Treatment delays and dose reductions: Though manageable, they complicate interpretation of real-world applicability.
- Single-nation scope: Less generalizable than multinational trials.
- No quality-of-life outcomes reported: Important for assessing chemotherapy's trade-offs.

4. Safety and efficacy of pembrolizumab, radiation therapy, and surgery versus radiation therapy and surgery for stage III soft tissue sarcoma of the extremity (SU2C-SARC032): an open-label, randomised clinical trial [15]

Study Design: Randomized phase II trial.

Strengths:

- Broad list of toxicities

Weaknesses:

- Phase II with a relatively small sample (64 patients in the intention to treat analysis)
- No improvement in OS
- Severy toxicity (56% of the treated patients had grade 3 or 4 AEs.)

5. A randomised phase II study on neo-adjuvant chemotherapy for 'high-risk' adult soft-tissue sarcoma[16]

Study Design: Phase II randomized trial of neoadjuvant doxorubicin/ifosfamide.

Strengths:

- Chemotherapy was feasible and didn't impair surgery.
- Long median follow-up (~7 years).

Weaknesses:

- Trial closed early due to poor accrual—underpowered for efficacy.
- No statistically significant survival benefit observed.

6. Adjuvant CYVADIC Chemotherapy for Adult Soft Tissue Sarcoma-Reduced Local Recurrence but No Improvement in Survival: A Study of the European Organization for Research and Treatment of Cancer Soft Tissue and Bone Sarcoma Group[17]

Study Design: Randomized, multicenter trial.

Strengths:

- Large cohort across 17 centers.
- Showed reduced local recurrence in chemo arm.
- Rigorous histological review process.

Weaknesses:

- No improvement in OS or DFS.
- CYVADIC regimen associated with significant toxicity.
- Older trial (design reflects limitations of era).

7. Intensified adjuvant IFADIC chemotherapy in combination with radiotherapy versus radiotherapy alone for soft tissue sarcoma: long-term follow-up of a prospective randomized feasibility trial[10]

Study Design: Randomized controlled trial

Strengths

- Long-term follow-up: Median of 97 months, which is valuable for evaluating recurrence and survival in cancer.
- Randomized controlled design: Patients were randomly assigned to treatment or control.
- Defined inclusion/exclusion criteria: Well-articulated eligibility and tumor grading.

Weaknesses

- Under-recruitment: Failed to reach planned sample size of 100 patients.
- Imbalance in group characteristics: Randomization did not stratify by tumor grade.
- No blinding: Open-label design.

8. Eastern Cooperative Oncology Group: A Comparison of Adjuvant Doxorubicin and Observation for Patients With Localized Soft Tissue Sarcoma[11]

Study Design: Randomized controlled trial

Strenghts

- Randomized design: Patients were randomly assigned to doxorubicin vs. observation.
- Multicenter study: Conducted within the ECOG framework, enhancing generalizability.
- Well-defined inclusion/exclusion criteria: Clear eligibility conditions and stratified randomization.

Weaknesses

- Small sample size and poor accrual: Only 30 evaluable patients (out of 47), reducing power to detect differences.
- Inclusion of varied tumor stages and sites: May obscure subgroup effects.
- No survival benefit shown: Result inconclusive, with a trend *against* doxorubicin in disease-free interval.

9. Vinorelbine and continuous low-dose cyclophosphamide as maintenance chemotherapy in patients with high-risk rhabdomyosarcoma (RMS 2005): a multicentre, open-label, randomised, phase 3 trial[18]

Study Design: Multicentre, open-label, randomised, phase 3 trial

Strenghts:

- Large, multicentric, international, Randomized Phase 3 trial with 371 patients — strong study design.
- Large follow-up (median follow-up 60.3 months) — allows for a robust observation of long-term outcomes.
- Detailed follow-up of toxicities — shows tolerability alongside efficacy.

Weaknesses:

- Open-label design, not blinded — this can introduce bias in clinician-reported outcomes and care delivery.
- Some crossover (1 patient continued maintenance despite assignment; 3 declined after randomization), which can confuse intent-to-treat analyses.
- Subgroup analyses were exploratory and not powered to detect differences — might produce false signals.

10. Safety and efficacy of regorafenib in patients with advanced soft tissue sarcoma (REGOSARC): a randomised, double-blind, placebo-controlled, phase 2 trial[19]

Study Design: Randomised, double-blind, Placebo-controlled phase 2 trial

Strengths:

- strong design
- Strict blinding — clinician, radiologists, pathologists blinded to treatment arm — reducing detection bias.
- Detailed safety profile — frequently grade 2 or greater toxicities were documented

Weaknesses:

- Large number of subgroups with small patient numbers — makes interpreting subgroup effects less reliable.
- Crossover after progression introduces potential bias in Overall Survival.

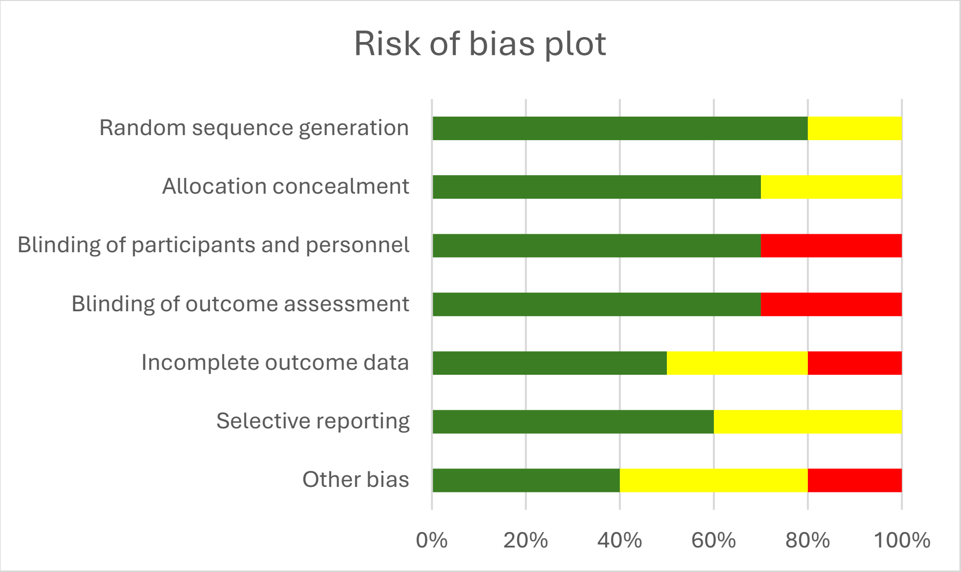

Supplementary Figure S 1
Risk of bias plot

1. Bonvalot S, Gronchi A, Le Pechoux C, Swallow CJ, Strauss D, Meeus P, et al. Preoperative radiotherapy plus surgery versus surgery alone for patients with primary retroperitoneal sarcoma (EORTC-62092: STRASS): a multicentre, open-label, randomised, phase 3 trial. Lancet Oncol. 2020;21(10):1366–77.
2. Judson I, Verweij J, Gelderblom H, Hartmann JT, Schoffski P, Blay JY, et al. Doxorubicin alone versus intensified doxorubicin plus ifosfamide for first-line treatment of advanced or metastatic soft-tissue sarcoma: a randomised controlled phase 3 trial. Lancet Oncol. 2014;15(4):415–23.
3. Issels RD, Lindner LH, Verweij J, Wessalowski R, Reichardt P, Wust P, et al. Effect of Neoadjuvant Chemotherapy Plus Regional Hyperthermia on Long-term Outcomes Among Patients With Localized High-Risk Soft Tissue Sarcoma: The EORTC 62961-ESHO 95 Randomized Clinical Trial. JAMA Oncol. 2018;4(4):483–92.

4. Berclaz LM, Jurinovic V, Burkhard-Meier A, Abdel-Rahman S, Albertsmeier M, Klein A, et al. Doxorubicin Plus Dacarbazine Versus Doxorubicin Plus Ifosfamide in Combination With Regional Hyperthermia in Patients With Advanced Leiomyosarcoma: A Propensity Score-Matched Analysis. Cancer Med. 2025;14(4):e70655.
5. Alvegard TA, Sigurdsson H, Mouridsen H, Solheim O, Unsgaard B, Ringborg U, et al. Adjuvant chemotherapy with doxorubicin in high-grade soft tissue sarcoma: a randomized trial of the Scandinavian Sarcoma Group. J Clin Oncol. 1989;7(10):1504–13.
6. Gronchi A, Palmerini E, Quagliuolo V, Martin Broto J, Lopez Pousa A, Grignani G, et al. Neoadjuvant Chemotherapy in High-Risk Soft Tissue Sarcomas: Final Results of a Randomized Trial From Italian (ISG), Spanish (GEIS), French (FSG), and Polish (PSG) Sarcoma Groups. J Clin Oncol. 2020;38(19):2178–86.
7. Pratt CB, Pappo AS, Gieser P, Jenkins JJ, Salzbergdagger A, Neff J, et al. Role of adjuvant chemotherapy in the treatment of surgically resected pediatric nonrhabdomyosarcomatous soft tissue sarcomas: A Pediatric Oncology Group Study. J Clin Oncol. 1999;17(4):1219.
8. Weiss AR, Chen YL, Scharschmidt TJ, Xue W, Gao Z, Black JO, et al. Outcomes After Preoperative Chemoradiation With or Without Pazopanib in Non-Rhabdomyosarcoma Soft Tissue Sarcoma: A Report From Children's Oncology Group and NRG Oncology. J Clin Oncol. 2023;41(31):4842–8.
9. Eilber FR, Giuliano AE, Huth JF, Morton DL. A randomized prospective trial using postoperative adjuvant chemotherapy (adriamycin) in high-grade extremity soft-tissue sarcoma. Am J Clin Oncol. 1988;11(1):39–45.
10. Fakhrai N, Ebm C, Kostler WJ, Jantsch M, Abdolvahab F, Dominkus M, et al. Intensified adjuvant IFADIC chemotherapy in combination with radiotherapy versus radiotherapy alone for soft tissue sarcoma: long-term follow-up of a prospective randomized feasibility trial. Wien Klin Wochenschr. 2010;122(21-22):614–9.
11. Lerner HJ, Amato DA, Savlov ED, DeWys WD, Mittleman A, Urtasun RC, et al. Eastern Cooperative Oncology Group: a comparison of adjuvant doxorubicin and observation for patients with localized soft tissue sarcoma. J Clin Oncol. 1987;5(4):613–7.
12. van der Graaf WT, Blay JY, Chawla SP, Kim DW, Bui-Nguyen B, Casali PG, et al. Pazopanib for metastatic soft-tissue sarcoma (PALETTE): a randomised, double-blind, placebo-controlled phase 3 trial. Lancet. 2012;379(9829):1879–86.
13. Woll PJ, Reichardt P, Le Cesne A, Bonvalot S, Azzarelli A, Hoekstra HJ, et al. Adjuvant chemotherapy with doxorubicin, ifosfamide, and lenograstim for resected soft-tissue sarcoma (EORTC 62931): a multicentre randomised controlled trial. Lancet Oncol. 2012;13(10):1045–54.
14. Frustaci S, Gherlinzoni F, De Paoli A, Bonetti M, Azzarelli A, Comandone A, et al. Adjuvant chemotherapy for adult soft tissue sarcomas of the extremities and girdles: results of the Italian randomized cooperative trial. J Clin Oncol. 2001;19(5):1238–47.
15. Mowery YM, Ballman KV, Hong AM, Schuetze SM, Wagner AJ, Monga V, et al. Safety and efficacy of pembrolizumab, radiation therapy, and surgery versus radiation therapy and surgery for stage III soft tissue sarcoma of the extremity (SU2C-SARC032): an open-label, randomised clinical trial. Lancet. 2024;404(10467):2053–64.
16. Gortzak E, Azzarelli A, Buesa J, Bramwell VH, van Coevorden F, van Geel AN, et al. A randomised phase II study on neo-adjuvant chemotherapy for 'high-risk' adult soft-tissue sarcoma. Eur J Cancer. 2001;37(9):1096–103.

17. Bramwell V, Rouesse J, Steward W, Santoro A, Schraffordt-Koops H, Buesa J, et al. Adjuvant CYVADIC chemotherapy for adult soft tissue sarcoma--reduced local recurrence but no improvement in survival: a study of the European Organization for Research and Treatment of Cancer Soft Tissue and Bone Sarcoma Group. J Clin Oncol. 1994;12(6):1137–49.
18. Bisogno G, De Salvo GL, Bergeron C, Gallego Melcon S, Merks JH, Kelsey A, et al. Vinorelbine and continuous low-dose cyclophosphamide as maintenance chemotherapy in patients with high-risk rhabdomyosarcoma (RMS 2005): a multicentre, open-label, randomised, phase 3 trial. Lancet Oncol. 2019;20(11):1566–75.
19. Mir O, Brodowicz T, Italiano A, Wallet J, Blay JY, Bertucci F, et al. Safety and efficacy of regorafenib in patients with advanced soft tissue sarcoma (REGOSARC): a randomised, double-blind, placebo-controlled, phase 2 trial. Lancet Oncol. 2016;17(12):1732–42.
